# Usability, Benefits, and Barriers Associated With Patients’ Access to Electronic Health Record–Integrated Telehealth in Hospitals in Riyadh: Qualitative Study

**DOI:** 10.2196/74011

**Published:** 2025-11-25

**Authors:** Dalia Alomar, Iliada Eleftheriou, Pauline Whelan, John Ainsworth

**Affiliations:** 1 Division of Informatics, Imaging and Data Sciences, Faculty of Biology, Medicine and Health, University of Manchester, Vaughan House, Portsmouth St, Manchester, M13 9GB, United Kingdom, 44 7917941877; 2 NIHR Manchester Biomedical Research Centre, Manchester University Hospitals NHS Foundation Trust, Manchester Academic Health Science Centre, Manchester, United Kingdom

**Keywords:** EHR-integrated telehealth, electronic health record, usability, patient engagement, remote health care, Riyadh hospitals, health care professionals, telehealth barriers, qualitative research

## Abstract

**Background:**

The integration of electronic health records (EHRs) with telehealth platforms represents a transformative approach in health care, providing critical accessibility and engagement solutions, especially during the COVID-19 pandemic. In Riyadh’s hospitals, the adoption of EHR-integrated telehealth has significantly increased and offers enhanced patient care options. However, there is a need to examine its continued relevance, effectiveness, and challenges in a postpandemic context.

**Objective:**

This research aimed to qualitatively investigate the usability, perceived benefits, and barriers to patients’ access to EHR-integrated telehealth from both patients and health care providers (HCPs) in a major Riyadh hospital.

**Methods:**

A qualitative research design was used, featuring semistructured interviews with 20 patients and 10 HCPs, selected through purposive sampling for their direct experience with EHR-integrated telehealth services at Sulaiman Al Habib Hospital in Riyadh. Thematic analysis, supported by NVivo 14 software, was used to analyze the transcriptions and extract themes related to usability, perceived benefits, and barriers.

**Results:**

The findings indicate that patients generally regard EHR-integrated telehealth positively, appreciating its navigability, convenience, and facilitation of remote health care interactions. Reported benefits included reduced physical visits, time savings, and more accessible follow-ups, contributing to greater continuity of care. However, significant barriers were identified, including technical challenges, lack of integration across hospital branches, absence of insurance payment linkages, and limited patient choice among providers. HCPs also expressed concerns over digital literacy gaps, the platform’s limitations for specialized and complex care, and technical disruptions impacting care delivery.

**Conclusions:**

EHR-integrated telehealth offers substantial potential to improve health care delivery in Riyadh’s hospitals by enhancing access, convenience, and patient engagement. However, maximizing these benefits in Saudi Arabia’s evolving health care landscape requires addressing identified barriers, particularly in platform stability, interbranch integration, insurance linkages, and patient support resources. Findings are grounded in a single-hospital sample and are intended to inform improvements in similar hospital settings in Saudi Arabia rather than national generalization.

## Introduction

### Background

The COVID-19 pandemic fundamentally transformed health care delivery, prompting rapid adoption of telehealth worldwide. In the United States, telehealth usage surged by 600% during the early months of the pandemic, reaching nearly 17,000 visits [1]. One health care organization reported that by transitioning 87% of its appointments to telehealth within 3 weeks, clinical volume decreased by only 15% [2]. Similarly, data from the Centers for Medicare and Medicaid Services indicated a rise in weekly telehealth visits from 13,000 before the pandemic to 1.7 million in April 2020 [3]. Compared with 2019, telehealth visits increased by more than 3000% by October 2020 [4]. Patients reported improved access and convenience, whereas providers emphasized time efficiency and enhanced communication [5].

Among the approaches that emerged during this period was the integration of electronic health records (EHRs) with telehealth platforms. EHR-integrated telehealth combines digital consultation capabilities with EHR functionalities, such as appointment scheduling, health record access, and test result management [6]. This integration is considered transformative, enabling streamlined access to clinical data, continuity of care, and improved patient–provider communication. Reported advantages include reducing travel time and costs, improving chronic disease follow-up, and extending care to underserved populations [7-9]. Globally, studies confirm that telehealth integrated with EHRs can improve satisfaction, care coordination, and efficiency [10-15]. For example, in Germany, integration reduced waiting times and facilitated chronic disease management, whereas telemonitoring has demonstrated benefits for conditions such as hypertension [16]. Nonetheless, persistent barriers remain, including disparities in access to technology, variable digital literacy, and privacy concerns.

In Saudi Arabia, pushing toward Vision 2030 digital health goals, telehealth adoption expanded rapidly during the pandemic and continues to play a central role in the country’s digital health transformation [17,18]. Leading hospitals in Riyadh have implemented EHR-integrated telehealth platforms, but existing Saudi research has primarily examined telehealth or EHRs in isolation, without integration [17,18]. In addition, limited research has simultaneously explored the perspectives of both patients and health care providers (HCPs) or examined operational barriers such as insurance integration, interoperability between hospital branches, and regulatory gaps. Given Saudi Arabia’s unique health care infrastructure, cultural context, and reliance on insurance coverage, these factors may influence adoption differently than in Western contexts. Addressing this research gap is crucial to inform sustainable, equitable digital health models both within Saudi Arabia and in comparable Gulf health systems. In addition, the focus on end-user experiences is particularly important. Patients provide insight into accessibility, convenience, and barriers to engagement, whereas HCPs contribute perspectives on workflow integration, service delivery, and system limitations. Exploring these experiences together offers a balanced understanding of how EHR-integrated telehealth is currently functioning in practice and where targeted improvements are most needed. In addition, this study emphasizes Riyadh’s role as a hub for advanced digital health initiatives, making it an ideal setting to generate insights with potential policy relevance across the Kingdom.

Conceptually, this study is guided by the Technology Acceptance Model (TAM), which posits that perceived ease of use and perceived usefulness are key determinants of EHR-integrated telehealth technology adoption [5], and by the principles of person-centered care, which emphasize tailoring health care services to patient needs and engagement. These frameworks informed the development of the research questions and findings about usability, perceived benefits, and barriers to EHR-integrated telehealth adoption in Saudi Arabia, as well as how it supports patient engagement and provider workflows.

### Objective and Research Questions

This study aimed to fill this research gap by qualitatively exploring the experiences of patients’ access to EHR-integrated telehealth from the perspectives of both patients and HCPs in Riyadh. Specifically, it investigated the usability of these services, the perceived benefits, and the challenges faced by patients. By addressing these aspects, the study sought to provide insights that will inform the optimization of telehealth services in Riyadh’s health care institutions and contribute to improving the accessibility and quality of health care delivery in the region. To achieve the study objectives, the following research questions were raised:

1. How do patients and HCPs perceive the usability of the EHR-integrated telehealth platform?
2. What benefits are reported in terms of health care accessibility, communication, and management?
3. What technical, organizational, and regulatory barriers constrain effective use of these services?
4. What improvements do patients and HCPs recommend to optimize EHR-integrated telehealth in Riyadh’s hospitals?

## Methods

### Study Design

This study used a qualitative research design, situated within a constructivist paradigm, to explore the usability, benefits, and challenges associated with patients’ access to EHR-integrated telehealth services in Riyadh hospitals. A qualitative approach was chosen for its capacity to generate rich, in-depth insights into the subjective experiences and perceptions of both patients and HCPs.

The first investigator (DA) collected data from November 2024 to February 2025 through semistructured, in-depth face-to-face interviews, which were conducted inside the Sulaiman Al Habib Hospital. While electronic interviews via the secure hospital’s telehealth platforms were available as part of the study context, all participants preferred in-person interviews, which provided opportunities for real-time clarification and deeper discussion of emerging themes. Interviews lasted between 50 and 70 minutes (mean 60, SD 7.8 min), allowing sufficient time for participants to reflect on their experiences and perceptions in detail. The semistructured format ensured consistency across participants while allowing for flexibility in questioning, enabling the researcher to probe further into emerging themes. The lead investigator (DA) maintained a reflexive stance throughout, considering her dual position as a health informatics researcher and health care professional, and regularly documented reflections in a field journal to mitigate potential biases.

### Research Setting

The study was conducted at Dr. Sulaiman Al Habib Hospital, a leading private health care group in Riyadh, known for its early and extensive adoption of telehealth services integrated within its EHR system, making it a suitable setting for this study. The hospital’s telehealth platform, the Al Habib App, provides patients with functionalities such as virtual consultations, appointment scheduling, prescription renewal, laboratory result viewing, and secure messaging. For HCPs, the system supports integrated documentation, access to patient medical records, and coordination across departments.

### Recruitment

Participants were recruited from Sulaiman Al Habib Hospital in Riyadh, which was selected because of its well-established and comprehensive EHR-integrated telehealth services. A purposive sampling strategy was used to capture insights from both patients and HCPs with direct experience using the platform. To ensure a range of perspectives, participants were selected based on their roles and engagement with telehealth services. The study included 10 HCPs from different specialties and departments and 20 patients with varying medical conditions and telehealth usage histories, ensuring the inclusion of individuals who interacted with the system in different capacities to provide a comprehensive understanding of its implementation and impact. Participant recruitment continued until thematic saturation was reached [19], where no new themes or codes emerged, indicating that the dataset sufficiently captured participants’ experiences. Prior literature suggests that between 20 and 30 interviews typically capture sufficient thematic variability for studies of this nature [19].

Eligible patient participants included individuals aged ≥18 years of all genders, medical conditions, and socioeconomic backgrounds and who have used EHR-integrated telehealth services at least once in the past year, ensuring their relevant experience with these services. Eligible HCP participants were selected to represent a range of specialties (general practice, obstetrics or gynecology, ophthalmology, eHealth and telehealth, and dentistry) and roles, including physicians, nurses, and administrative staff involved in telehealth for at least 6 months to ensure their sufficient experience in using it. The recruitment process involved direct invitations extended to HCPs through department heads and professional networks, while patients were contacted based on hospital telehealth records, as well as through recruitment advertisements or brochures placed in hospital waiting areas. This strategy ensured a diverse and relevant sample of participants with varying levels of interaction with EHR-integrated telehealth, capturing a broad spectrum of experiences regarding its usability and impact. The research team acknowledges that this approach may have excluded less-engaged or digitally disadvantaged individuals. This limitation was partly mitigated by including patients with varied levels of telehealth familiarity and by encouraging referrals from clinicians to reach less active users. Before the start of any interviews, individual interviews with participants were implemented in the hospital to answer any participant questions.

### Interview Guide

The interview guide for this study was structured to capture in-depth insights from both patients and HCPs regarding EHR-integrated telehealth services in Riyadh’s hospitals. It was piloted with 2 participants (ie, 1 patient and 1 HCP) to ensure clarity, relevance, and sequencing of questions. Minor refinements were made based on feedback before commencing full data collection. The final guide included questions that covered 6 key areas: (1) introduction and background information, where patients shared their demographics and medical conditions, while HCPs shared their specific role-related context; (2) usability, exploring experiences with platform navigation, appointment scheduling, and communication tools; (3) perceived benefits, capturing positive impacts on health care management, accessibility, and patient engagement; (4) perceived barriers and challenges, identifying obstacles such as technical issues, privacy concerns, and digital literacy limitations; (5) general experience and suggestions, which allowed participants to reflect on their satisfaction and provide recommendations for improvement; and (6) contextual factors, examining the influence of socioeconomic, organizational, and regulatory factors on telehealth access and use. This semistructured design ensured consistency across interviews while allowing flexibility for follow-up questions, supporting a comprehensive understanding of EHR-integrated telehealth’s impact on health care delivery in this context. Multimedia Appendix 1 shows questions asked to patients and HCP participants in the interviews. The questions were developed in English, translated into Arabic, and reviewed by an independent bilingual health informatics specialist to ensure both linguistic accuracy and conceptual equivalence. This ensured that all questions were comprehensible to participants while minimizing translation bias.

### Qualitative Analysis

The qualitative analysis followed a systematic process to identify key themes related to EHR-integrated telehealth through comprehensive coding and thematic content analysis. Interviews were audio recorded and transcribed verbatim. All transcripts and audio files were translated from Arabic into English by a professional translator. The average transcript length was approximately 14 to 16 pages (mean 15, SD 2 pages). Data analysis was carried out using Qualitative Research Software (QSR International NVivo software version 14), following Braun and Clarke’s thematic content analysis approach [20].

Two investigators (DA and JA) conducted a thematic content analysis of the interview transcripts to identify emerging patterns. After independently coding the first 5 interviews, an initial codebook has been developed. This preliminary codebook was then used to recode the first 5 interviews and code all remaining interviews, with periodic cross-checks and regular meetings held between research team members to discuss and resolve any coding discrepancies and refine the codebook to integrate new emerging themes, ensure consistency, and maintain reliability. Triangulation was achieved through comparing perspectives of patients and HCPs, and reflexivity was maintained through the investigators’ audit trail and reflexive notes documenting their positionality and assumptions during analysis.

Study design and interview analysis were reported using the Consolidated Criteria for Reporting Qualitative Studies 32-item checklist (Checklist 1) [21].

In this study, rigor was ensured through multiple strategies, including intercoder reliability, triangulation of patient and HCP perspectives, and systematic use of NVivo to manage data. Reflexivity was addressed by acknowledging that the first investigator (DA), as a health informatics researcher with prior experience in telehealth evaluation, has shaped the data collection and interpretation. Reflexive notes were maintained to critically reflect on positionality, interview dynamics, and potential biases. Translation was another important consideration. Although all interviews were conducted in Arabic, the analysis was carried out on English translations. To mitigate risks of meaning loss and enhance data quality, after all interviews were professionally translated into English by an independent translator, these translations were reviewed by the first investigator and a bilingual health care researcher familiar with the Saudi context.

### Ethical Considerations

Ethics approval was secured from the University of Manchester Research Ethics Committee 2 (reference 2024-20964-36807), along with institutional review board approval from the Sulaiman Al Habib Hospital administration for the procedures, participant recruitment, and interview questions. Verbal informed consent was obtained before the start of any interview, per the guidelines of the University of Manchester Research Ethics Committee 2. All transcripts and audio files were stored without personal identifiers on a secure, password-protected server to ensure participant confidentiality. No compensations were provided to participants for their participations in this study.

## Results

### Participants

In total, 30 participants (ie, 20 patients and 10 HCPs) have been interviewed. Regarding patient demographics, as presented in Table 1, 40% (8/20) were aged between 20 and 30 years, 50% (10/20) were aged between 31 and 40 years, and 10% (2/20) were aged between 41 and 50 years. Gender distribution was balanced, with 50% (10/20) males and 50% (10/20) females. In terms of medical conditions, 85% (17/20) of patients reported being in good health with no chronic diseases, whereas 15% (3/20) reported having chronic conditions, such as diabetes or high blood pressure. For the duration of EHR-integrated telehealth usage, 25% (5/20) of patients had used the service for less than a year, 45% (9/20) had used the service for 1 to 2 years, and 30% (6/20) had used the service for 3 to 5 years. In 2023, 35% of patients (7/20) did not use EHR-integrated telehealth services, 45% (9/20) used them 1 to 4 times, 15% (3/20) used them 5 to 10 times, and 5% (1/20) used them 11 to 12 times.

**Table 1.** Participant demographics.

Characteristics			Value, n (%)
Patients (n=20)			
	Age (y)		
		20‐30	8 (40)
		31‐40	10 (50)
		41‐50	2 (10)
	Sex		
		Male	10 (50)
		Female	10 (50)
	Medical condition		
		Good (ie, no disease)	17 (85)
		Chronic disease (ie, diabetes, high blood pressure)	3 (15)
	Duration of EHR^a^-integrated telehealth utilization (y)		
		<1	5 (25)
		1‐2	9 (45)
		3‐5	6 (30)
	Number of times EHR-integrated telehealth was used in the past year (2023)		
		None	7 (35)
		1‐4	9 (45)
		5‐10	3 (15)
		11‐12	1 (5)
HCPs^b^ (n=10)			
	Position and role		
		General practitioner: provides in-person and remote consultations, diagnoses medical conditions, manages chronic disease follow-up, prescribes medications, and coordinates tests and treatment plans	4 (40)
		Consultant obstetrician/gynecologist: manages women’s health, including diagnosis, treatment, pregnancy follow-up, and performing childbirth and gynecological surgeries	2 (20)
		Consultant ophthalmologist: specializes in diagnosing and treating eye diseases and conditions	1 (10)
		Doctor at eHealth and telehealth department: delivers remote consultations, diagnoses, prescribes treatments, manages lab and radiology orders, and coordinates with multidisciplinary teams for comprehensive patient care	2 (20)
		Dentist: handles inquiries and provides dental consultations through online medical services	1 (10)
	Duration of use of EHR-integrated telehealth with your patients (y)		
		<1	1 (10)
		1‐3	2 (20)
		≥4 (since COVID-19)	7 (70)

a EHR: electronic health record.

b HCP: health care provider.

For HCP demographics, participants were distributed across several positions and related roles: 4 (40%) were general practitioners who provided both in-person and remote consultations, managed chronic disease follow-ups, prescribed medications, and coordinated patient testing and treatment plans. Two participants (20%) were consultant obstetricians/gynecologists who specialized in women’s health, including diagnosis, treatment, and pregnancy follow-up, and conducting childbirth and other gynecological surgeries. One participant (10%) was a consultant ophthalmologist focused on treating eye diseases, whereas 2 participants (20%) worked in the eHealth and telehealth department, providing remote consultations, managing laboratory and radiology requests, and coordinating care with multidisciplinary teams. Finally, 1 participant (10%) was a dentist handling inquiries and providing dental consultations through online services. In terms of their experience with EHR-integrated telehealth with their patients, 1 HCP (10%) had used it for less than a year, 2 (20%) had used it for 1 to 3 years, and 7 (70%) had been using it for 4 years or more since the COVID-19 pandemic.

Participants varied in age and telehealth experience. While formal education or household income measures were not systematically collected, multiple patients commented on socioeconomic and literacy factors that influenced telehealth experiences. Most patients reported that cost did not prevent their use of the app, but a minority raised affordability concerns (eg, “the prices of services are not cheap”). Several respondents also noted that older adults or those unfamiliar with apps required assistance or found navigation harder (eg, “some older people may have difficulty using techniques”). Internet reliability and basic technical skills emerged repeatedly as determinants of effective use.

The analysis ultimately organized findings around 4 major themes, from perspectives of both patients and HCPs, related to EHR-integrated telehealth, including usability, perceived benefits, barriers and challenges, and general experiences and suggestions for improvements. Table 2 summarizes the number of coded references assigned to each major theme across all interviews. These frequency counts reflect how often ideas were mentioned in the dataset (salience of issues in the corpus) rather than the number of participants who endorsed a theme. The analytic approach combined iterative codebook development with dual coding. Recruitment continued until the 26th interview, where no novel codes were identified across consecutive interviews and the codebook stabilized [19], indicating that the dataset sufficiently captured participants’ experiences. The final 4 interviews yielded only confirmatory evidence of existing themes. Both patient and HCP subgroups reached saturation independently, ensuring coverage across perspectives.

**Table 2.** Frequency of identified themes related to access and utilization of EHR^a^-integrated telehealth.

Theme	Frequency values^b^, n	
	Patients	HCPs^c^
Usability	80	42
Perceived benefits	117	80
Barriers to and challenges of access and utilization	93	47
General experiences and suggestions for improvements	69	57

a EHR: electronic health record.

b Frequency was calculated as the total number of references related to the group of codes of each theme by interviewees.

c HCP: health care provider.

### Perspectives on Usability of EHR-Integrated Telehealth

#### Overview

In the discussion of perspectives of both patients and HCPs about the patients’ experiences and usability of telehealth features integrated within the EHRs framework in Sulaiman Al Habib Hospital, interviewees discussed several ways in which this platform could form this usability, including (1) user interaction and experience and (2) technical performance and communication issues.

#### User Interaction and Experience

Across patient interviews, the Al Habib platform was broadly perceived as easy to access and navigate for routine tasks (eg, booking, viewing records, and prescription refills). Patients frequently described straightforward appointment booking and ready access to prior visit notes and test results, emphasizing time savings and convenience. For example, 1 patient said:

Very good. Easy access to medical information such as laboratory scans, future appointments, bookings, and medical statements. The information is available at all times and has easy access.

#### HCPs’ Perspective and Cross-Group Comparison

HCPs generally echoed patients’ reports about the platform’s accessibility and functionality for routine care, noting that the app supports efficient appointment management and medication refills. However, clinicians emphasized the platform’s limitations for complex diagnostic work and specialist assessment, which are areas where remote workflows were felt to be less appropriate. One HCP remarked:

In general, the services available through the Al Habib application meet the needs of patients, especially those who have minor cases or need periodic follow-up. However, there are some patients who try to use the service to diagnose complex conditions, which may need a physical examination, which is difficult to achieve via remote service. Some patients need specialized matters, which cannot be answered through Life Care app*.*

[HCP 3]

Overall, patients and HCPs agreed that the app is well suited for minor conditions and follow-up but less so for complex or examination-dependent cases.

#### Technical Performance and Communication

Communication features (notably video) were valued when they worked, but instability (ie, poor video/audio quality, dropped calls, or the need to switch to telephone) was a frequent barrier that reduced clinical usefulness for some encounters. One patient described intermittent failures:

The video calls often freeze, lose sound, or the camera not working properly. Then, it’s hard to explain my symptoms when the connection is bad, forcing the doctor to call me on the phone instead.

HCPs similarly reported that poor video quality prevented adequate visual assessment in some cases.

### Perceived Benefits

#### Overview

When interviewees were asked about the perceived benefits of EHR-integrated telehealth for healthcare delivery, patient care, and access to health care services, they consistently emphasized three domains of benefit from EHR-integrated telehealth: (1) accessibility and convenience, (2) healthcare management and support, and (3) patient satisfaction.

#### Accessibility and Convenience

Patients reported that telehealth saved significant time, effort, and transportation costs, particularly for routine check-ups, prescription refills, or minor concerns. They valued the ability to consult from home or work without disrupting daily commitments or arranging transportation. One patient explained:

Comfort and saving time and effort. I do not need to get out of the house... including the access to medical consultations and prescriptions, and delivering medicines to the patient.

HCPs echoed these views, noting that remote consultations reduced unnecessary hospital visits and allowed patients to access care from home or workplaces. As one HCP observed:

The app makes patients able to follow their health condition and conduct consultations from anywhere, which offers great convenience.

Both groups agreed that convenience was one of telehealth’s strongest advantages, especially for patients with mobility challenges or those living farther from the hospital.

#### Health Care Management and Support

Patients highlighted how access to medical records, test results, and treatment histories improved their ability to monitor health conditions and adhere to care plans. For example, one patient stated:

Telehealth has helped me compare old and new medical tests, making it easier to track the progress of my health condition and better manage treatment.

HCPs similarly noted that telehealth supported adherence and continuity of care, especially for patients with chronic diseases. One HCP explained:

The service enhanced patients' understanding of their health condition and increased their commitment to treatment and follow-up.

Together, these perspectives suggest that the platform encouraged more active and sustained patient engagement in managing health.

#### Patient Satisfaction

Patients expressed high satisfaction with telehealth, attributing it to ease of access, improved communication, and the ability to manage health care needs without frequent in-person visits. One patient shared:

Using telehealth has increased my satisfaction because I feel I have easy access to healthcare whenever I need it.

HCPs reported observing similar satisfaction among their patients, pointing to reduced travel and positive feedback through reviews and interactions. As one HCP put it:

Patients are very satisfied with the service; it has provided them with comfort and reduced unnecessary hospital visits.

Overall, satisfaction for both patients and HCPs stemmed from a combination of convenience, improved health management, and more responsive communication with providers.

### Barriers and Challenges

#### Overview

When patients and HCP interviewees were asked about barriers and challenges in patients’ access to and utilization of EHR-based telehealth, they identified multiple barriers, grouped into six main domains including technical and functional barriers, health care service delivery issues, integration and coordination problems, prescription, medication, and analyses results challenges, patient privacy and security concerns, and contextual, organizational, or regulatory factors.

#### Technical and Functional Barriers

Patients frequently described frustrations with appointment booking, long waiting times, and poor video quality, often linked to internet instability or lack of timely technical support. These issues disrupted consultations and delayed access to care, especially for older adults less comfortable with digital tools. As one patient shared:

The video calls often freeze, lose sound, or the camera not working properly. Then, it’s hard to explain my symptoms when the connection is bad.

HCPs similarly reported that connectivity problems and patients’ limited technical skills complicated consultations. One HCP noted:

Patients with limited tech skills struggle to navigate the app, which adds to our workload as we need to guide them through basic steps.

Together, these findings from both patients and HCPs perspectives underscore the importance of reliable infrastructure and digital literacy in supporting telehealth usability.

#### Health Care Service Delivery Issues

Patients raised concerns about limited doctor availability, lack of specialist consultations, and the inability to select a preferred physician for continuity of care. Some also noted frustration at not being able to secure sick leave documentation via telehealth. One patient explained:

I would prefer to consult with the same doctor each time, but the app doesn’t give me that choice.

HCPs echoed these issues, highlighting that certain specialties (eg, gynecology, dermatology) are poorly suited for remote care and that patients’ dissatisfaction with sick leave requests often drove them back to in-person visits. As one HCP stated:

Telehealth doesn’t suit all specialties; for example, physical assessments in areas like gynecology are hard to examine remotely

[HCP 2]

Both perspectives highlight the limits of telehealth as a universal substitute, emphasizing the need for targeted use depending on condition and specialty.

### Integration and Coordination Problems

#### Overview

Patients reported inefficiencies due to poor coordination between hospital branches, lack of insurance payment links, and weak integration with pharmacies, which often forced them to repeat tests or pay out-of-pocket. One patient described:

...only the problem is not linking the payment for the virtual visit to the insurance...the patient may refrain from remote consultation completely...

HCPs confirmed that the absence of insurance integration and pharmacy linkage undermined continuity of care and patient satisfaction. One HCP explained:

Health insurance does not cover the virtual consultation, laboratory tests, and basic services without additional approvals from insurance.

Both groups pointed to system-level gaps that reduce efficiency and discourage ongoing telehealth use.

#### Prescription, medication, and results challenges

Patients noted delays in accessing lab results and inconsistencies in prescription transmission to pharmacies, which often required extra calls or visits. One patient reported:

Sometimes the doctor writes the prescription but it does not get to the pharmacy properly, forcing me to repeat the operation or visit the hospital in person.

HCPs added that the lack of effective triage systems within the platform complicated workflow, as urgent and non-urgent cases were not differentiated. One HCP remarked:

One of the main challenges is the lack of an effective triage system to identify patients who need specialized or general consultation.

Both groups show how incomplete integration impedes timely follow-up and efficient care delivery.

### Patient Privacy and Security Concerns

#### Overview

Interestingly, most patients expressed confidence in the platform’s security, reporting no major worries about privacy breaches. One patient stated:

No, I don't have any concerns about the privacy or security of my medical information when using the platform.

HCPs likewise confirmed that privacy concerns were rarely raised. One explained:

I did not notice any concern among patients about the privacy of their medical information. They are all confident that their data is secure.

This alignment suggests that strong institutional reputation may build trust, even in digital health adoption.

#### Contextual, Organizational, and Regulatory Factors

Patients generally felt that socio-economic background did not limit access, though insurance requirements and regulatory gaps were seen as barriers. One patient reflected:

I think the app is available to everyone and there are no social or economic barriers to its use... perhaps medical insurance, as sometimes it requires communication with the insurance provider*.*

HCPs, however, emphasized organizational and regulatory shortcomings, including the exclusion of patients under 17 years old. As one HCP noted:

“The application does not serve patients under 17, which may constitute a barrier for some mothers for their young children or infants.

Together, these perspectives illustrate how broader structural factors constrain the inclusivity and long-term sustainability of telehealth.

### General Experiences and Suggestions for Improvements

Thematic analysis provided some other findings related to patients’ general experience and their suggestions for improvement as a result of their access and utilization of EHR-based telehealth. Patients generally reported positive experiences with the EHR-integrated telehealth platform, valuing its convenience, timeliness, and efficiency. Satisfaction was high, with many highlighting quick access to prescriptions and consultations as key benefits. One patient noted:

I am very satisfied with how quickly I can get my prescriptions and consult with my doctor through the app.

At the same time, some patients emphasized the need for ongoing improvements, particularly in technical support, customer service, and expanded service offerings (eg, sick leave and specialist access), stating that:

The application needs to be improved in technical aspects, such as full support for video calls and chat to include photos to be considered a real clinic.

As another patient explained:

Overall, while satisfaction was high, patients balanced praise with constructive feedback, demonstrating both appreciation and expectation for continued platform development.

Looking ahead, patients expressed strong willingness to continue using telehealth services in the future, especially for routine consultations and follow-ups. This preference was linked to perceived convenience and time savings. One patient summarized:

I would definitely prefer using the app for future appointments; it saves me so much time and facilitates access to health services.

This enthusiasm suggests that telehealth is viewed not only as a temporary solution but as a sustainable option for ongoing health care management.

From the perspective of HCPs, the platform was also seen as beneficial in improving patients’ access and convenience. HCPs confirmed high levels of patient satisfaction but pointed to persistent challenges, such as technical glitches, difficulties managing complex cases remotely, and limited organizational support. One HCP remarked:

Telehealth has improved access for patients, but there are limitations in handling complex cases that really need in-person consultations.

In addition, HCPs offered several suggestions for improvement, including stronger technical infrastructure, an effective triage system, expanded record integration across branches, and more targeted training for both staff and patients. As one HCP explained:

Both staff and patients would benefit from additional training to enhance telehealth literacy and platform usability. Establishing a dedicated telehealth team focused solely on app-based patients could reduce wait times and improve care quality.

Finally, HCPs raised concerns about the regulatory and insurance frameworks, noting that clearer guidelines and broader coverage would enhance compliance, protect patient data, and ensure equitable access. One physician emphasized:

It is important to improve policies related to insurance and expand its coverage of remote care services. Regarding sick leave, there must be organizational aspects to this in order to determine whether or not a patient is eligible.

Taken together, patients and HCPs converged on the need for technical, organizational, and policy improvements to support sustainable, effective, and trusted telehealth delivery.

## Discussion

### Principal Findings

Using a qualitative analysis of semistructured interviews, this study explored the usability, perceived benefits, barriers, and overall experiences with EHR-integrated telehealth services in Riyadh’s hospitals from both patient and HCP perspectives. Four key themes emerged: usability, perceived benefits, barriers and challenges, and general experiences and suggestions for improvement. Findings suggest that while EHR-integrated telehealth enhances accessibility and convenience, persistent challenges in technical functionality, service delivery, integration, and regulation limit its full potential. These insights contribute to understanding telehealth in the Saudi context, which has received less attention compared to Western settings.

The usability of the telehealth platform was highlighted as a key strength. Both patients and HCPs found the system intuitive and user-friendly, consistent with existing literature on telehealth adoption [2]. This supports the TAM, where perceived ease of use influences positive attitudes toward technology and sustained engagement [5]. At the same time, persistent technical issues (eg, video quality and connectivity) contradict TAM’s expectation that ease of use consistently drives acceptance, showing that infrastructural weaknesses can undermine adoption even when the interface is simple. These contradictions highlight the need for stronger digital infrastructure in Saudi Arabia to fully realize TAM’s potential pathways.

For perceived benefits, patients emphasized reduced travel time, improved flexibility, and timely access to care, whereas HCPs highlighted greater patient engagement and adherence. These findings are consistent with international evidence on telehealth’s ability to reduce logistical burdens and support chronic disease management [22]. Importantly, telehealth’s capacity to foster ongoing communication also aligns with person-centered care, which prioritizes continuity and patient engagement. However, not all findings were positive: for certain specialties requiring physical examinations (eg, orthopedics and dermatology), telehealth fell short. This contradiction underscores that while telehealth enhances patient-centeredness for routine care, it may compromise quality in complex or specialist care contexts, requiring hybrid approaches.

Regarding barriers, service delivery, and contextual differences, barriers identified (eg, technical failures, limited availability of doctors, lack of specialist access, poor insurance integration, and regulatory gaps) mirror challenges reported in other Gulf settings [7,23]. However, they diverge from many Western contexts where insurance integration and regulatory frameworks are more advanced [17]. In relation to person-centered care, patients and HCPs also emphasized difficulties with continuity of care, such as inability to choose specific doctors and delays in result integration across hospital branches. These findings reinforce that in Saudi Arabia, organizational and policy-level issues, rather than patient trust or motivation, pose the greatest barriers to telehealth. Addressing these issues is vital for sustainable expansion.

Overall, both patients and HCPs reported high satisfaction with telehealth but stressed the importance of improvements. Suggested priorities included better technical support, more robust triage systems, training for digital literacy, and integration of insurance and medical records across hospital branches. These recommendations are consistent with global calls for continuous quality improvement in telehealth [11,24,25]. Importantly, the emphasis on insurance integration and regulatory reform reflects region-specific priorities not commonly observed in Western telehealth studies [26].

Importantly, while the findings illustrate important opportunities and challenges, they should not be interpreted as broadly representative of Saudi Arabia as a whole. The study focused on a single hospital with advanced EHR–telehealth integration, which may not reflect the full diversity of health care settings in the country. Nevertheless, the findings provide valuable insights into emerging trends and patient–provider perspectives in one of Saudi Arabia’s leading hospitals, offering lessons that can inform national strategies for scaling telehealth.

### Strengths and Limitations

This study’s primary strength lies in its dual-perspective approach, capturing insights from both patients and HCPs to provide a balanced and comprehensive understanding of EHR-integrated telehealth’s usability, benefits, and challenges. Conducted in the postpandemic period, the study is timely, reflecting the increased reliance on digital health care solutions in Saudi Arabia and offering findings highly relevant for shaping future health care strategies. Moreover, the study’s focus on Saudi Arabia highlights specific regional needs, such as geographic accessibility and system integration, providing actionable insights tailored to the Saudi health care landscape and potentially influencing policy developments for digital health care services across the region.

However, this study has several limitations that should be acknowledged. First, its geographic scope was limited to a single hospital in Riyadh, which may reduce the transferability of findings across Saudi Arabia’s diverse regions, where health care access, infrastructure, and digital adoption levels can vary. In addition, the purposive sample of 30 participants, while sufficient for achieving thematic saturation, cannot support broad generalizations, and the perspectives reported should be interpreted as illustrative rather than representative. Second, potential sources of bias must be considered. Social desirability bias may have led participants to emphasize positive experiences, while interviewer positionality as a health informatics researcher could have influenced the framing of questions or interpretation of responses. Translation of transcripts from Arabic to English, although reviewed by an independent translator, may also have introduced subtle nuances affecting meaning. Third, recruitment through hospital records and brochures may have disproportionately included patients who were already more engaged with health care services and excluded those less digitally literate or less inclined to access telehealth, thereby limiting the diversity of perspectives captured. In addition, while the qualitative methodology was valuable in generating rich, in-depth insights, it does not provide quantitative data needed to establish statistically significant trends in telehealth usage and satisfaction. As with any qualitative research, participants’ willingness to share their experiences was influenced by comfort level, interview setting, and trust in the research process, which may have shaped the narratives obtained. Finally, the short-term nature of this study restricts insights into long-term patient engagement, sustained provider adaptation, and the effectiveness of proposed improvements. Taken together, these limitations highlight the importance of future studies with a broader scope, mixed-method approaches, and longitudinal designs to validate and extend these findings across different health care settings in Saudi Arabia.

### Conclusions

This study offers an in-depth exploration of patient and HCP perspectives on EHR-integrated telehealth services in Riyadh, contributing context-specific insights to the broader global evidence base. While many of the reported benefits (such as enhanced accessibility, convenience, and patient engagement) resonate with international findings, this study underscores distinctive challenges in the Saudi context, including limited insurance integration, fragmented coordination across hospital branches, and regulatory gaps. These findings suggest that the value of telehealth in Saudi Arabia lies not in novelty, but in its potential to be tailored and scaled within the country’s unique health care ecosystem.

Moving beyond technical fixes, several actionable pathways emerge. First, policy alignment is critical. Clear regulatory frameworks and mandatory insurance reimbursement for telehealth consultations would reduce financial and administrative barriers. The Ministry of Health also should issue standardized telehealth regulatory guidelines covering privacy, security, and sick leave certification, reducing inconsistencies across hospitals. It is also important to create direct digital payment links between telehealth platforms and insurance providers, allowing real-time claim approvals within the app rather than requiring manual follow-up. Second, organizational investment is needed to expand specialist availability for remote care, establish dedicated telehealth teams, and implement structured triage protocols to prioritize urgent cases. Third, capacity building for both patients and HCPs through targeted digital literacy programs and staff training could improve navigation, communication, and trust in the platform. Fourth, technological integration should prioritize interoperability across hospital branches and pharmacies, ensuring continuity of care and seamless access to medical records and prescriptions.

Future research should evaluate hybrid care models that combine the convenience of telehealth with in-person consultations for conditions requiring physical examination, whereas longitudinal studies could clarify its impact on chronic disease management, patient outcomes, and sustained engagement. In addition, comparative research across Saudi regions would help determine how local cultural and infrastructural factors shape telehealth adoption. As Saudi Arabia advances its Vision 2030 digital health agenda, the lessons drawn from this study can inform the design of more inclusive, patient-centered, and policy-aligned telehealth solutions. By embedding telehealth within a robust regulatory, technological, and organizational framework, the country can move beyond short-term pandemic-driven adoption toward a sustainable model of digitally enabled health care.

## Supplementary material

10.2196/74011 Multimedia Appendix 1 Interview questions.

10.2196/74011 Checklist 1 COREQ 32-item checklist.

## Abbreviations

EHR: electronic health record
HCP: health care provider
TAM: Technology Acceptance Model

## References

[1] Carlson JL, Goldstein R (2020). Using the electronic health record to conduct adolescent telehealth visits in the time of COVID-19. J Adolesc Health.

[2] Maheswaranathan M, Chu P, Johannemann A, Criscione-Schreiber L, Clowse M, Leverenz DL (2022). The impact of the COVID-19 pandemic and telemedicine implementation on practice patterns and electronic health record utilization in an academic rheumatology practice. J Clin Rheumatol.

[3] Koonin LM, Hoots B, Tsang CA (2020). Trends in the use of telehealth during the emergence of the COVID-19 pandemic - United States, January-March 2020. MMWR Morb Mortal Wkly Rep.

[4] Omboni S, Padwal RS, Alessa T (2022). The worldwide impact of telemedicine during COVID-19: current evidence and recommendations for the future. Connect Health.

[5] Ghaddar S, Vatcheva KP, Alvarado SG, Mykyta L (2020). Understanding the intention to use telehealth services in underserved Hispanic border communities: cross-sectional study. J Med Internet Res.

[6] Zhang X, Saltman R (2022). Impact of electronic health record interoperability on telehealth service outcomes. JMIR Med Inform.

[7] Lawson DW, Stolwyk RJ, Ponsford JL, Baker KS, Tran J, Wong D (2022). Acceptability of telehealth in post-stroke memory rehabilitation: a qualitative analysis. Neuropsychol Rehabil.

[8] Madu R (2020). Using technology to reduce barriers in community health: an intersection between broadband access EHR & telehealth adoption in federally qualified health centers [PhD dissertation]. https://digitalcommons.library.tmc.edu/uthsph_dissertsopen/127.

[9] Wiley KK, Mendonca E, Blackburn J, Menachemi N, Groot MD, Vest JR (2022). Quantifying electronic health record data quality in telehealth and office-based diabetes care. Appl Clin Inform.

[10] McLendon SF (2017). Interactive video telehealth models to improve access to diabetes specialty care and education in the rural setting: a systematic review. Diabetes Spectr.

[11] Ashwood JS, Mehrotra A, Cowling D, Uscher-Pines L (2017). Direct-to-consumer telehealth may increase access to care but does not decrease spending. Health Aff (Millwood).

[12] Ndwabe H, Basu A, Mohammed J (2024). Post pandemic analysis on comprehensive utilization of telehealth and telemedicine. Clinical eHealth.

[13] Bitar H, Alismail S (2021). The role of eHealth, telehealth, and telemedicine for chronic disease patients during COVID-19 pandemic: a rapid systematic review. Digit Health.

[14] Ben-Assuli O (2022). Measuring the cost-effectiveness of using telehealth for diabetes management: a narrative review of methods and findings. Int J Med Inform.

[15] Cushing M (2022). What is telemedicine, telehealth, and teletriage. Vet Clin North Am Small Anim Pract.

[16] Omboni S (2021). Connected health: in the right place at the right time. Connect Health Telemed.

[17] Moussa FL, Moussa ML, Alharbi HA (2023). Telehealth readiness of healthcare providers during COVID-19 pandemic in Saudi Arabia. Healthcare (Basel).

[18] Alotaibi HF, Ghazi SS, Asraf NO, Al Asmari ZS (2023). Patients’ perceptions of telehealth video visits experience in primary healthcare setting, Saudi Arabia. J Family Med Prim Care.

[19] Guest G, Bunce A, Johnson L (2006). How many interviews are enough? An experiment with data saturation and variability. Field Methods.

[20] Braun V, Clarke V (2006). Using thematic analysis in psychology. Qual Res Psychol.

[21] Tong A, Sainsbury P, Craig J (2007). Consolidated criteria for reporting qualitative research (COREQ): a 32-item checklist for interviews and focus groups. Int J Qual Health Care.

[22] Edwards L, Thomas C, Gregory A (2014). Are people with chronic diseases interested in using telehealth? A cross-sectional postal survey. J Med Internet Res.

[23] Sinha Gregory N, Shukla AP, Noel JJ (2023). The feasibility, acceptability, and usability of telehealth visits. Front Med (Lausanne).

[24] Al Baalharith I, Al Sherim M, Almutairi SHG, Albaqami ASA (2022). Telehealth and transformation of nursing care in Saudi Arabia: a systematic review. Int J Telemed Appl.

[25] Verma R, Krishnamurti T, Ray KN (2020). Parent perspectives on family-centered pediatric electronic consultations: qualitative study. J Med Internet Res.

[26] Alharbi RA (2023). Awareness, practices, attitudes, and barriers of telehealth In Saudi Arabia. J Pak Med Assoc.

