# Supplementary material for: Usability, Benefits, and Barriers Associated With Patients’ Access to Electronic Health Record–Integrated Telehealth in Hospitals in Riyadh: Qualitative Study
Source: JMIR Form Res. 2025 Nov 25;9:e74011. doi: 10.2196/74011 (PMC12646287; doi:10.2196/74011)
Supplement: Multimedia Appendix 1 [file formative-v9-e74011-s001.docx]

**Usability, benefits, and barriers associated with patient access to EHRs-integrated telehealth in Riyadh's hospitals**

**Patient Interview Questions**

- **Introduction and Demographic Information:**

1. Can you please tell me a bit about yourself? (e.g., age, gender, medical condition, frequency of hospital visits)

2. How long have you been using telehealth services through the hospital’s EHR system?

3. How often have you used telehealth services integrated with EHRs in the past year?

- **Usability:**

4. How would you describe your experience navigating/accessing the telehealth platform integrated with the EHR system?

5. How easy or difficult do you find scheduling appointments through the EHR system for telehealth consultations?

6. Are there any specific aspects of the telehealth platform that you find particularly user-friendly or challenging?

7. How would you rate the communication features (e.g., video calls, chat) within the telehealth platform? Why?

- **Perceived Benefits:**

8. What are the main benefits you have experienced from using telehealth services through the EHR system? (e.g., convenience, reduced travel time, improved access to healthcare)

9. How has telehealth impacted your ability to manage your health condition or treatment plan?

10. Do you feel that telehealth has improved your communication with healthcare providers? How**?**

11. Do you feel that telehealth has improved your healthcare engagement? How**?**

12. How has accessing telehealth services impacted your overall healthcare experience and satisfaction?

- **Perceived Barriers and Challenges:**

13. What challenges have you faced when using telehealth services through the EHR system?

14. Are there any specific features or functionalities of the telehealth platform that you find difficult to use?

15. Are there any specific challenges you face in accessing or using telehealth services? (e.g., internet connectivity, lack of technical skills)

16. Have you encountered any technical issues while using telehealth services through the EHR system? If so, please describe them.

17. Do you have any concerns regarding the privacy and security of your medical information when using telehealth services?

18. How have these challenges affected your overall experience with telehealth services?

- **General Experience and Suggestions:**

19. Overall, how satisfied are you with the telehealth services provided through the hospital’s EHR system?

20. What suggestions do you have for improving the telehealth services integrated with the EHR system?

21. Would you prefer to continue using telehealth services in the future? Why or why not?

22. Is there anything else you would like to share about your experience with telehealth services?

- **Specific Contextual Factors**

23. How do you think your socioeconomic background (sex, age, nationality…) has influenced your ability to access and use telehealth services?

24. Have there been any organizational or regulatory factors at the hospital that affected your use of telehealth services?

**Usability, benefits, and barriers associated with patient access to EHRs-integrated telehealth in Riyadh's hospitals**

**Healthcare Providers (HCPs) Interview Questions**

- **Introduction and Background:**

1. Can you please tell me a bit about your role and responsibilities at the hospital?
2. How long have your patients been involved in using telehealth services through the hospital’s EHR system?

- **Usability:**

1. From your perspective, how would you describe your patients usability of the telehealth platform integrated with the EHR system?
2. What are your thoughts on the patient ease of use and navigation of the telehealth features within the EHR framework?
3. How user-friendly do your patients find the EHRs-integrated telehealth platform for managing their consultations and appointments?
4. In your patient experience, how effective are the communication tools (e.g., video conferencing, chat, messaging) provided by the EHRs-integrated telehealth platform for consultations?

- **Perceived Benefits:**

1. What are the main benefits your patients have observed from using telehealth services through the EHR system for their care?
2. Do you feel that EHR-integrated telehealth services have enhanced the accessibility and quality of care for your patients? How?
3. In what ways has EHR-integrated telehealth improved patient healthcare engagement and management from your perspective?
4. Can you share any specific examples of how EHR-integrated telehealth has enhanced care coordination or patient outcomes in your practice?

- **Barriers and Challenges:**

1. What challenges have your patient encountered when accessing telehealth services through the EHR system?
2. Are there any specific features or functionalities of the EHRs integrated-telehealth platform that your patients find difficult or inefficient to use?
3. Are there any technical issues or limitations with the telehealth platform that your patients have observed?
4. Have you noticed any concerns among patients regarding privacy or security when using telehealth services through the EHR system?
5. How have these challenges affected their healthcare engagement or their ability to receive care through telehealth services?

- **General Experience and Suggestions:**

1. Overall, how satisfied are your patients with the telehealth services provided through the hospital’s EHR system?
2. What improvements or enhancements would you recommend to optimize the telehealth services integrated with the EHR system for patients?
3. Is there anything else you would like to share about experience of your patients with EHR-integrated telehealth services or any additional insights relevant to this study?

- **Contextual Factors:**

21. What organizational or cultural factors have influenced your patient access to and utilize EHR-integrated telehealth services in your hospital?

22. From your perspective, what regulatory or policy considerations are important for patient successful access to EHR-integrated telehealth?
